# High mitochondrial DNA sequence divergence in New Guinea *Carabdytes* stream beetles and the taxonomist’s dilemma when other evidence is kind of subtle… (and collecting localities are far far away)

**DOI:** 10.3897/zookeys.247.3812

**Published:** 2012-11-30

**Authors:** Andre Skale, Rene Tänzler, Lars Hendrich, Michael Balke

**Affiliations:** 1 Blücherstraße 46, 95030 Hof/ Saale, Germany; 2 Department of Entomology, Zoological State Collection, Munich, 81247 Germany; 3 GeoBioCenter, Ludwig-Maximilians-University, Munich, Germany

**Keywords:** Coleoptera, integrative taxonomy, cryptic species, DNA sequencing, DNA barcoding

## Abstract

*Carabdytes upin tindige* **ssp. n.** is described from the Arfak Mountains Bird’s Head Indonesian Papua. It is morphologically very similar to *Carabdytes upin upin* Balke et al. 1992 known from eastern Indonesian Papua eastward to the western limits of the Papuan Peninsula of Papua New Guinea. For 726 bp at the 3’ end of the mitochondrial *cox1* gene the subspecies differ by 8.1–9.2% uncorrected *p*-distance. However we also document considerable *cox1* divergence within *Carabdytes upin upin*. We find few diagnostic positions in the nuclear genes argenine kinase as well as elongation factor 1 alpha that suggest there are indeed two isolated groups of *Carabdytes* but evidence in elongation factor 1 alpha is not unambiguous. We decided to highlight this phenomenon of ambiguous evidence for ongoing/just attained speciation by describing a subspecies. We argue that such cases are actually common once mitochondrial sequence data are routinely added to the taxonomist’s toolkit and sometimes simply adding data from few nuclear genes will not suffice the solve taxonomic riddles. Here detailed population genetic investigations would be required – for which sufficient numbers of specimens from a sufficiently wide geographical sampling might be nearly impossible to acquire.

## Introduction

**Figure 1.**
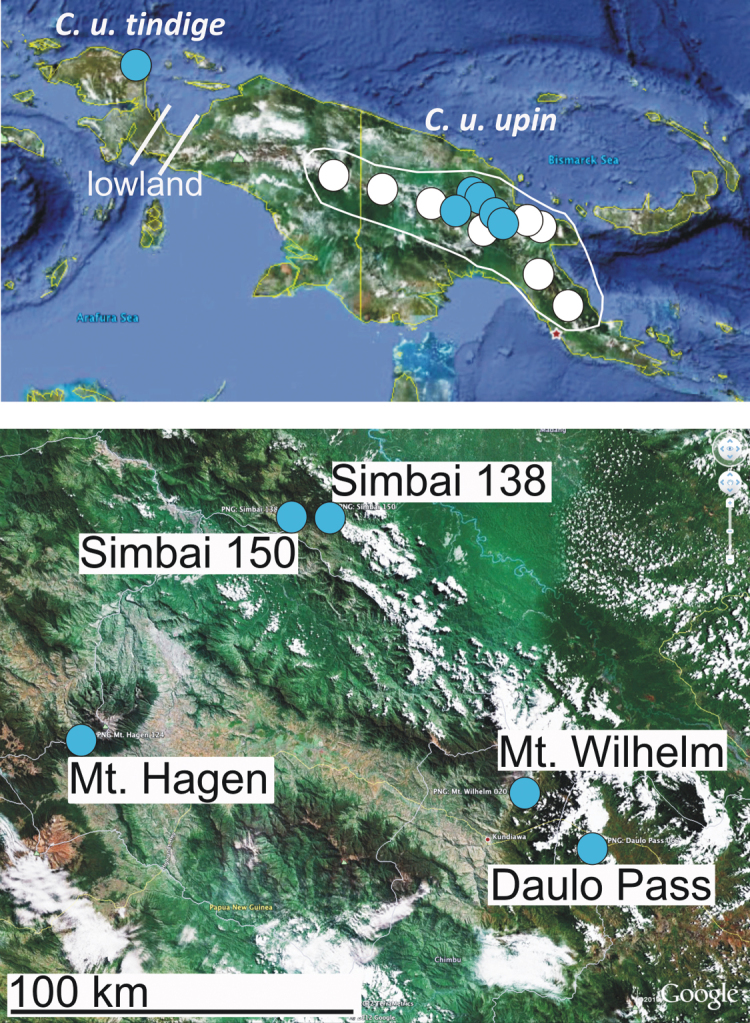
Distribution of *Carabdytes upin*, blue dots = sequenced specimens, white dots = other records. Lowland gap indicated by bars in the Bird’s neck. Below, detailed map of central Papua New Guinea with localities for sequenced specimens.

*Carabdytes* Balke et al., 1992, is a genus of New Guinea Colymbetinae diving beetles which to date only contains *Carabdytes upin* (Balke et al. 1992; Balke 2001). The species inhabits fast flowing, cold mountain rivers, where the beetles hide under large stones at the edge, but still in the water. The beetles also inhabit smaller, shaded, low order streams where they also tend to hide under stones or creep about in small stream pools and between stones in the stream bed. We also collected the species from deep, high altitude blackwater *Sphagnum* pools on peat, *c*. 2800–3400 m high (PNG, Kumul Lodge, see below). The beetles are dorso-ventrally flattened, and with their long legs with only few swimming hairs, and the basally constricted pronotum rather resemble ground beetles than other diving beetles (Fig. 3). Given its highly specialized, higher altitude-related ecology, it is surprising that the species is comparably widespread (Fig. 1), the easternmost localities in the Wau area of Papua New Guinea and the westernmost localities in Indonesian Papuan highlands being roughly 800 kilometers apart.

Recent molecular phylogenetic analyses revealed that *Carabdytes upin* belongs to an isolated clade of the Colymbetinae (Balke et al. 2007, 2009) which also contains several Oceanian *Rhantus* species such as *Rhantus novaecaledoniae* Balfour-Browne, 1944 and *Rhantus alutaceus* Fauvel, 1883, from New Caledonia. These species should be probably all assigned to *Carabdytes*, but the redefinition of *Carabdytes* will be possible only when a robust, global analyses of the Colymbetinae is finished (Balke et al. in prep.).

During an extensive survey across the island of New Guinea, we obtained *Carabdytes* samples from several new localities, pushing its range boundary approximately 700 kilometers westward to the Bird’s Head peninsula (Fig. 1). The fresh tissue was used for DNA purification and sequencing to study intraspecific variation. Here, we report surprisingly high mitochondrial DNA divergence in *Carabdytes upin*. We present evidence from nuclear protein coding genes and morphology that the Bird’s Head beetles might belong to a different species. We also describe the taxonomist’s dilemma when there is some evidence for the presence of cryptic species but perhaps not enough and there is no straightforward solution as the required additional localities are very remote and extremely difficult to visit. We argue this scenario might be not so rare, and new technology eagerly awaited by the traditional taxonomist does not always provide a fast and complete solution of the “old problems”.

## Material and methods

Beetles were preserved in 95% ethanol and flight muscle tissue was used for DNA purification. The laboratory methods employed are detailed on our DNA laboratory wiki: http://zsm-entomology.de/wiki/The_Beetle_D_N_A_Lab . PCR conditions with Mango Taq (Bioline) were for *cox1*: (primers: Jerry/Pat, Simon et al. 1994) 1’ 94°C – 40× (30s 94°C – 30s 47°C – 1’ 72°C) – 10’ 72°C; for Elongation Factor 1α (EF1α): (primers: efs372/efa754, McKenna and Farrell 2005; Normark et al. 1999) 5’ 95°C – 8× (30s 95°C – 1’ 58°C – 1’ 72°C – 30s 95°C – 1’ 58°C – 1’ 72°C – 30s 95°C – 1’ 58°C – 1’ 72°C) – 18× (30s 95°C – 1’42°C – 1’ 72°C); for Arginine Kinase: (primers: AK183F/AK939R, Wild and Maddison 2008) 3’ 94°C – 35× (30s 94°C – 30s 53°C – 1’ 72°C) – 10’ 72°C.

We use GARLI V.0.951 (Zwickl 2006) with default settings (using the GTR model of evolution with parameter estimation) to obtain a maximum likelihood tree of the *cox1* data. The SpeciesIdentifier module of TaxonDNA software v.1.6.2 was used to study the genetic divergences in our dataset and to cluster sequences at different preset thresholds using uncorrected *p*-distances (Meier et al. 2006; http://code.google.com/p/taxondna/ ). SpeciesIdentifier accounts for threshold violations according to triangle inequity (i.e., when the divergence between A – B and B – C is 3% or less, but A – C exceeds 3%, then A, B and C would still be grouped into one 3% cluster by Taxon DNA. We routinely use 3% as a preset threshold, as this value captures species boundaries comparably well for Dytiscidae (Hendrich et al. 2010).

Digital images were taken with a Nikon D3X with a Voigtländer Apo Lanthar 90 mm attached to a bellows; fitted to a custom built macro rail (image steps used: 0.4 mm). Image stacks were aligned and assembled with the computer software Helicon Focus 4.77^TM^.

**Institutional abbreviations:**

**CSH** Coll. Andre Skale, Hof/Saale, Germany

**MZB** Museum Zoologicum Bogoriense, LIPI, Cibinong, Indonesia

**NMW** Naturhistorisches Museum Wien, Austria

**ZSM** Zoologische Staatssammlung München, Germany

**Other abbreviation:**

**PNG** Papua New Guinea

Locality data for specimens of *Carabdytes upin* studied for this paper (* sequence data available, see Table 1):

**Papua New Guinea**

* Papua New Guinea: Simbu, Mt Wilhelm, lower lake from Keglsugl, 3500–3700 m, 23.ix.2002, ca. 05.53.733S, 145.02.742E, Balke & Sagata leg. (PNG 020) (ZSM)

* Papua New Guinea: Eastern Highlands, Goroka, Daulo Pass, 2500m, 19.v.2006, 06.02.432S, 145.13.333E, John & Balke leg. (PNG 67) (ZSM)

Papua New Guinea: Southern Highlands, Sopulkul, 30–35Km NE Mendi, from swamp that drains into stream, 2679m, 16.vi.2006, 06.02.944S, 143.46.485E, John leg. (PNG 79) (no DNA sequence data) (ZSM)

* Papua New Guinea: Enga, Kumul Lodge @ foot of Mt Hagen, 2700m, 5.xii.2006, 05.47.548S, 143.58.761E, Balke & Kinibel leg. (PNG 124) (ZSM)

* Papua New Guinea: Western Highlands, Simbai, 1800–2000m, 1.iii.2007, 05.14.276S, 144.28.741E, Kinibel leg. (PNG 138) (ZSM)

* Papua New Guinea: Western Highlands, Simbai area, 2500m, 8.iii.2007, 05.14.202S, 144.33.651E, Kinibel leg. (PNG 150) (ZSM)

**Indonesia: Papua**

Jayawijaya Mts., Aipomek-Diruemna, 2600m, 3.ix.1992, 04.26S, 139.57E, Balke leg. (no DNA sequence data) (NMW)

**Indonesia: West Papua**

* Bird’s Head, Manokwari, Mokwam (Siyoubrig), 1400–1800m, 24.–28.II.2007, 01.06.26S, 133.54.41E, Skale leg. (CSH, MZB, ZSM)

**Table 1.** Sequenced *Carabdytes* specimens and EMBL accession numbers.

		***cox1***	**EF1α**	**ARK**
*Carabdytes upin tindige* MB 3084	Papua: Arfak	HF558675	HF558686	HF558698
*Carabdytes upin* MB 3328	PNG 138: Simbai	HF558676	HF558687	HF558699
*Carabdytes upin* MB 3354	PNG 067: Daulo Pass	HF558677	HF558688	HF558700
*Carabdytes upin* MB3452	PNG 150: Simbai	HF558678	HF558689	
*Carabdytes upin* MB3453	PNG 150: Simbai	HF558679	HF558690	
*Carabdytes upin* MB3454	PNG 150: Simbai	HF558680	HF558691	
*Carabdytes upin* MB3455	PNG 150: Simbai	HF558681	HF558692	
*Carabdytes upin* MB3045	PNG 124: Mt. Hagen	HF558682	HF558693	HF558701
*Carabdytes upin* MB4316	PNG 067: Daulo Pass	HF558683	HF558694	
*Carabdytes upin* MB4317	PNG 138: Simbai	HF558684	HF558695	HF558702
*Carabdytes upin* MB4318	PNG 138: Simbai	HF558685	HF558696	HF558703
*Carabdytes upin* MB0306	PNG 020: Mt. Wilhelm	FN263070.1	HF558697	

## Results

### Analysis of genetic and morphological variation in *Carabdytes upin*

**Figure 2.**
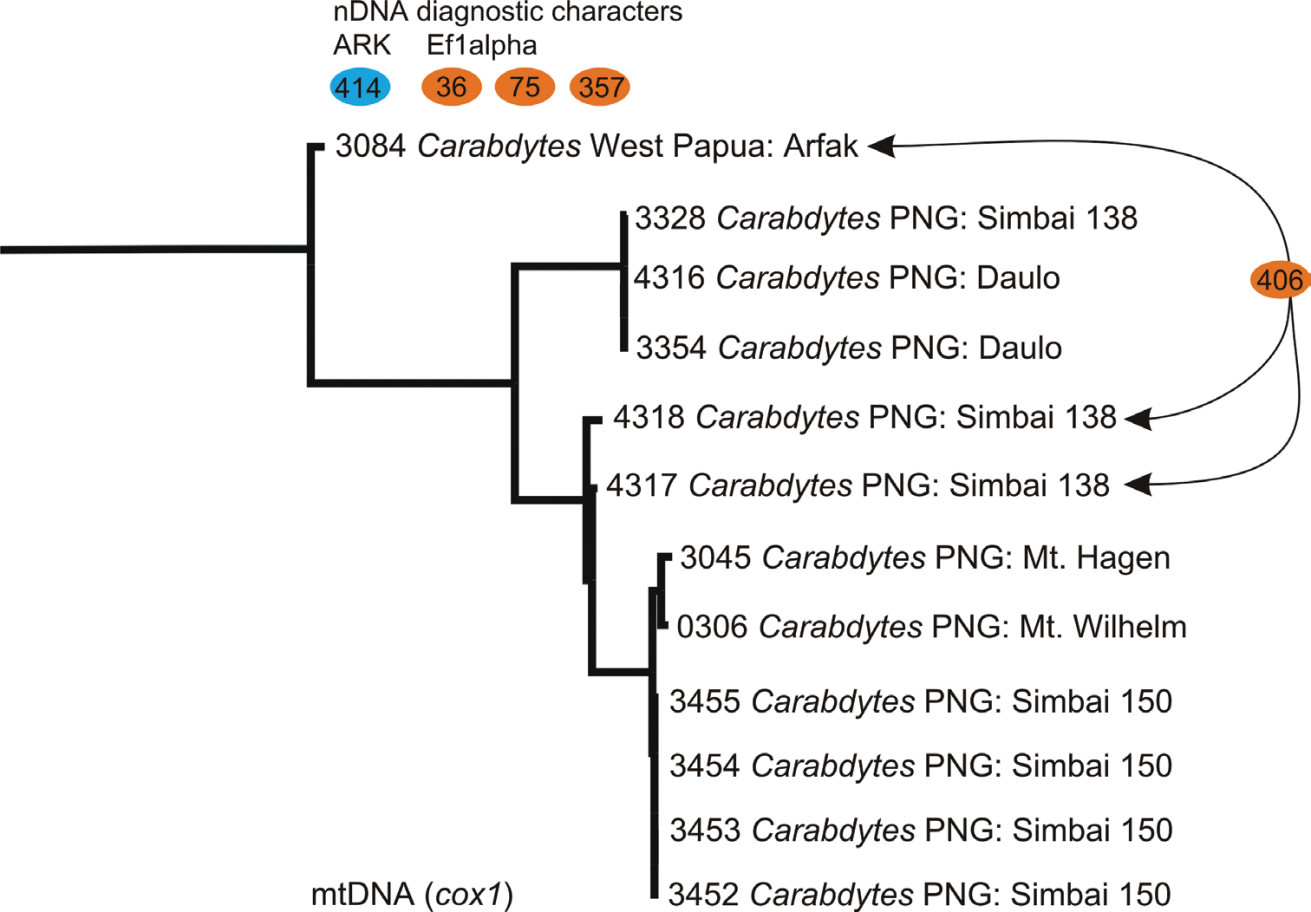
Maximum likelihood tree based on *cox1* data, the tree was rooted with *Rhantus guadalcanalensis* Balke (pruned here), colored buttons represent diagnostic nuclear DNA characters and their positions in our sequence alignment (there are found characters supporting the node between *Carabdytes* 3084 and all others, and one that is shared between 3084 as well as the two specimens 4317 & 4318 in Simbai 138) .

***Elongation factor 1 alpha*.** We obtained a 555 bp fragment in the 5’ region of EF1α for all specimens shown in Figure 2. There are 3 diagnostic nucleotide substitutions for a specimen from the Bird’s Head (MB3084, see Fig. 1) (positions 36, 75, and 357, all 3^rd^ codon positions) delineating this specimen from the other *Carabdytes*.

Specimens MB3084 (Bird’s Head) and MB4317, 4318 (PNG: Simbai) share a non synonymous substitution in position 406 (1^st^ codon position).

***Arginine Kinase*.** We obtained 656 bp of sequence data for 6 individuals of *Carabdytes* (Table 1). There was one diagnostic character, a 3^rd^ codon substitution in position 414 of our alignment. This character delineates the Bird’s Head specimen from the other *Carabdytes*. The sequences are otherwise identical.

***Cytochrome c oxidase 1*.** We obtained a 726 bp fragment at the 3’ of *cox1* for 12 individuals of *Carabdytes* (Table 1). Sequence data were surprisingly divergent, although most of the samples all originate from one major region in eastern New Guinea (Figs 1, 2). Uncorrected *p*-distances were 0–9.23%. There are 29 unambiguous diagnostic characters delineating the Bird’s Head specimen from the other *Carabdytes*, all of them in 3^rd^ codon positions.

The clade MB3328 / MB3354 / MB4316 has 20 diagnostic characters (and two 1^st^ codon substitutions resulting in amino acid change, pos. 316 and 415 in our alignment) and MB0306 / MB 3045 / MB3452–55 has 5 diagnostic characters (Fig. 2).

***Cluster analysis*.** At 3% preset threshold, SpeciesIdentifier finds four *cox1* clusters, which agree with the three main lineages of the tree in Fig. 2 (MB4317 and MB4318 form one cluster). For the nuclear markers, all data only form a single cluster at 3%.

**Figure 3.**
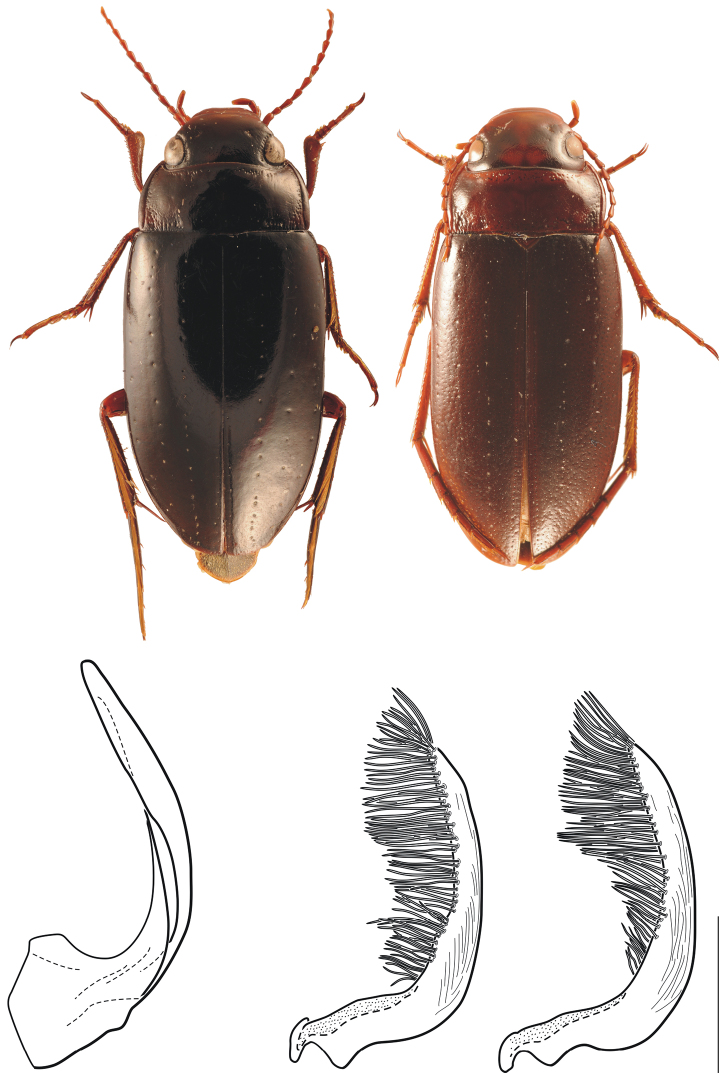
Morphological characters, above habitus, left *Carabdytes upin tindige* from the Bird’s Head (12.0 mm long), right *Carabdytes upin upin* from Papua: Aipomek area (12.9 mm long); below left median lobe of aedeagus in lateral view of *Carabdytes upin tindige* (*Carabdytes upin upin* is identical), its paramere, right, paramere of *Carabdytes upin upin* from Papua: Aipomek area. Scale for genitalia is 0.1 mm.

***Morphology*.**Four specimens were available from the Bird’s Head for morphological study. The distinguishing feature between these specimens and *Carabdytes upin* from eastern localities in New Guinea is: Pronotum and elytra conspicuously shining with very indistinct punctuation (the elytra have a conspicuous coarse punctuation, especially on the apical half, in eastern *Carabdytes upin*) (Fig. 3). Specimens of *Carabdytes upin* studied for this comparison come from the localities mentioned above, covering most of its range (no specimens studied from Huon Peninsula and Wau). The specimens from Simbai (locality PNG138) have an intermediate elytral punctation, with only few coarse punctures on the apical part, while specimens from Simbai (loc. PNG150) which is less than 20 kilometers apart (Fig. 1) are coarsely punctate. The Simbai localities both have specimens with attached sequence data.

## Taxonomic treatment

For *Carabdytes upin*, we do suggest to flag the Bird’s Head beetles with a subspecies name, assuming the combined, congruent observations described above are evidence for longer periods of interrupted gene flow. We suggest the use of a subspecies name to stimulate further investigation to verify or falsify this hypothesis.

### Carabdytes upin tindige ssp.n.

http://species-id.net/wiki/Carabdytes_upin_tindige

#### Type locality.

West Papua, Arfak Mountains, Siyoubrig , 01°06.26'S, 133°54.41'E.

#### Description.

**Holotype:** ♂ (MZB): Indonesia, West Papua, Arfak Mountains, stream near Siyoubrig 01°06.26'S, 133°54.41'E, 1400–1800 m, 24. –28.II.2007, leg. A. Weigel. **Paratypes:** 2♂♂ 1 ♀ (CSH, ZSM): same locality data as holotype (the female was sequenced).

**Habitus** as in Fig. 3; total length: 11.6–12.0 mm; total width: 5.3–5.5 mm. Dark brown to almost black; labrum, lateral margin of pronotum and all body appendages paler reddish brown; elongate.

#### Colour.

Head black, labrum reddish brown, clypeus with indistinctly reddish colour almost reaching eyes; head with indistinctly reddish patch on frons. Pronotum black, with indistinctly median reddish patch, posterior angles reddish. Ventral surface blackish. Venter dark brown.

**Structures.** Head with fine, sparse punctation interspersed with coarser punctures between eyes and behind anterior clypeal margin. Pronotum shining, posterior angles with irregular, coarse punctures, lateral margin very strongly. Elytra shining with very indistinctly punctures; each elytra with four rows of coarser, moderately arranged punctures. Lateral wing of metaventrite broad and tongue-shaped; outer margin slightly sinuate; last abdominal ventrite medially emarginate. Legs long and slender.

**Male.** Pro- and mesotarsal claws of similar structure; anterior and posterior claws moderately long and evenly curved; Median lobe of aedeagus relatively slender (Fig. 3, shape in *Carabdytes upin upin* is identical); paramere slender, with distinctly longitudinal striation; setation more or less long (Fig. 3), the setation might be basally shorter in some specimens of *Carabdytes upin upin* (Fig. 3), but this difference does not appear constant.

#### Diagnosis.

Distinguished from *Carabdytes upin upin* through the molecular and morphological characters mentioned in the results section under “morphology”. With *Carabdytes upin upin* there is no overall size difference (10.1–12.2 mm).

#### Habitat.

**Figure 4.**
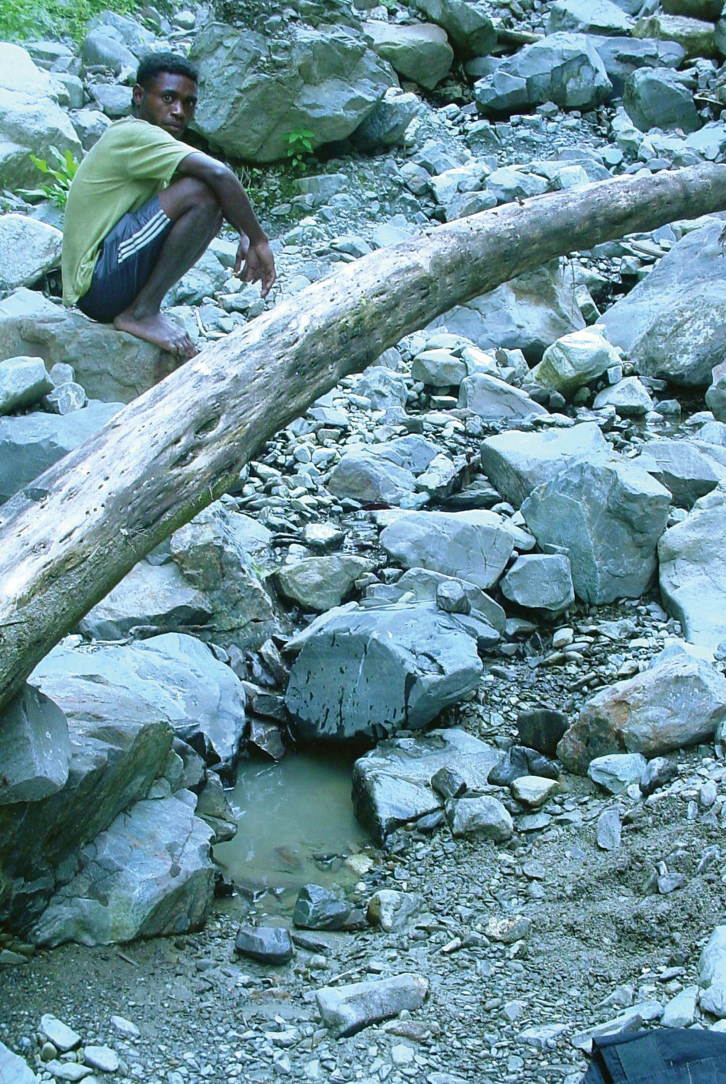
Type locality of *Carabdytes upin tindige* ssp.n.

Two individuals collected with an aquatic net from the rough gravel at the edge of a stream bed, the stream was rather dry at the time of collection (Fig. 4). The species co-occured with *Platynectes* spp., *Exocelina* (=*Papuadytes*) spp. and *Hydraena cristatigena* Jäch & Diaz. Two exemplars were collected with the help of a light trap, approx. 50 m away from the stream.

#### Distribution.

So far known only from the type locality (Figs 1, 4).

#### Etymology.

In loving memory of Samkris “Kris” Tindige, relentless conservationist in Papua, who left us too early. The beetles were collected in the stream bed very close to a birdwatching guesthouse set up by Kris and Shita Prativi above Siyoubrig village.

## Discussion

Here, we document mitochondrial DNA divergence of up to 9% within *Carabdytes upin*. Our samples mainly originate from the core range of this species, from eastern New Guinea. One specimen from the Bird’s Head Peninsula in the west of New Guinea, about 700 km west of the next known locality for *Carabdytes upin*, is well separated geographically from other populations. It is also most divergent genetically. The mountain regions between the known localities are understudied, but some (wider Wamena area eastwards to Diruemna; Nabire area up to Enarotalia; Cyclops Mountains near Jayapura) have specifically been screened for diving beetles. *Carabdytes upin* was not yet collected there. The vast expanse of karst as well as tropical lowland in the Bird’s neck region, roughly from Lake Yamur westwards to Arfak Mountains, offers few obvious habitats for *Carabdytes upin*, with Wandammen Peninsula as a potential stepping stone (though the species was not yet detected there) (Fig. 1, “lowland”).

Intraspecific mitochondrial *cox1* divergences >3 % are considered high in Dytiscidae. For the Australian fauna, largest intraspecific distances reported by Hendrich et al. (2010) were well below that (median 1.25%, mean 1.94%, SD 2.37%), and average distances even lower (median 0.50%, mean 0.71%, SD 0.80%). However, there are exceptions. Morphologically identical populations of *Copelatus* diving beetles from northern South America diverge up to 8% in *cox1*, with strong geographical signal (Balke et al. 2008). However the authors acknowledged that additional investigation was certainly warranted to understand how many species there really are. Such additional investigations were conducted here for *Carabdytes upin*, in this case sequencing of nDNA loci.

Cryptic species are apparently more common and phylogenetically more widespread than assumed previously (Pfenninger and Schwenk 2007). The use of molecular methods, namely extensive mitochondrial DNA sequencing or barcoding, routinely uncovers strong genetic subdivision among morphologically highly similar or indiscernible populations. In many cases, this even concerns species in well-studied faunas which were supposedly widespread and abundant, and not necessarily understudied faunas or taxa only (e.g. Hebert et al. 2004). However, detection of unusually high mitochondrial divergence *per se* does not satisfyingly support cryptic species hypotheses and additional lines of evidence should be followed, no matter which species concept is used (Hawlitschek et al. 2011; Tänzler et al. 2012).

In the morphologically highly similar *Carabdytes upin*, we find geographical separation and high *cox1* divergence. In the nDNA marker Arginine Kinase, we find one diagnostic character for the Bird’s Head beetle, in elongation factor 1 alpha(EF1α) there are three, but all of these are synonymous substitutions not altering the amino acid sequence and thus protein derived from the nucleotide sequence. For EF1α, there is another substitution, but this one is shared between the Bird’s Head specimen and two specimens from eastern New Guinea (Simbai, PNG138, MB4317 & 4318) (Figs 1, 2). A third specimen from the Simbai PNG138 locality has the same EF1α genotype as all other *Carabdytes upin*. Within *Carabdytes upin* from eastern New Guinea, we also observe considerable mtDNA variation, up to 7.7% (Table 2). Importantly, this also concerns close localities such as Simbai PNG138 and PNG150, less than 10 km apart. Moreover, haplotypes from locality Simbai PNG138 also differ around 7% from each other.

**Table 2.** Uncorrected *cox1 p*-distances for *Carabdytes* specimens (* the new subspecies from the Bird’s Head).

		**1**	**2**	**3**	**4**	**5**	**6**	**7**	**8**	**9**	**10**	**11**	**12**
0306 Carabdytes PNG Mt Wilhelm	1	__											
3045 Carabdytes PNG Mt Hagen	2	0.010	__										
3452 Carabdytes PNG150 Simbai	3	0.010	0.011	__									
3453 Carabdytes PNG150 Simbai	4	0.010	0.011	0.000	__								
3454 Carabdytes PNG150 Simbai	5	0.010	0.011	0.000	0.000	__							
3455 Carabdytes PNG150 Simbai	6	0.010	0.011	0.000	0.000	0.000	__						
3328 Carabdytes PNG138 Simbai	7	0.077	0.077	0.073	0.073	0.073	0.073	__					
4316 Carabdytes PNG067 Daulo	8	0.076	0.076	0.072	0.072	0.072	0.072	0.001	__				
3354 Carabdytes PNG067 Daulo	9	0.076	0.076	0.072	0.072	0.072	0.072	0.001	0.000	__			
4318 Carabdytes PNG138 Simbai	10	0.040	0.047	0.039	0.039	0.039	0.039	0.068	0.066	0.066	__		
* 3084 Carabdytes West Papua Arfak	11	0.087	0.080	0.086	0.086	0.086	0.086	0.093	0.091	0.091	0.090	__	
4317 Carabdytes PNG138 Simbai	12	0.032	0.039	0.030	0.030	0.030	0.030	0.070	0.069	0.069	0.014	0.084	__

Thus, there is considerable *cox1* variation, as expected in running water organisms, or species in highly fragmented habitats in general (Abellan et al. 2007; Engelhardt et al. 2008), but here this variation is apparently only partially structured geographically. Most Melanesian running water beetles exhibit pronounced endemism and microendemism (Balke 1999), and species are usually similar morphologically yet with clear differentiation in genital structure and often in terms of body size, color and fine sculpture. This is not the case in *Carabdytes upin*. There are diagnostic nDNA characters delineating the Bird’s Head sample and other *Carabdytes upin*, as well as geographic separation. This case would now warrant in-depth study of population level processes, but it is not possible to collect the amount of specimens from the higher number of localities required for such approaches (see Abellan et al. 2007 for an adequate sampling design).

What are the practical implications from the beetle taxonomist’s point? Mitochondrial DNA variation alone does not provide sufficient evidence. While divergence between eastern and western localities is high, such is divergence even within *one* of the eastern localities, as well. Thus, we tried to find other *congruent* evidence that might indicate presence of a cryptic species. In the nuclear genes Arginine Kinase and elongation factor 1 alpha, we count a total of 4 diagnostic characters (Fig. 2). This is additional evidence, combined with the high mtDNA divergence, of interrupted gene flow over longer periods. It is interesting to note that two specimens from Simbai (locality PNG138) which also diverge highly from other *Carabdytes upin* share 1 diagnostic EF1α position with the Bird’s Head specimen. As described above, the Simbai (PNG138) specimens are morphologically intermediate between the Bird’s Head specimens and other studied *Carabdytes upin*. Overall molecular evidence suggests they belong to the eastern clade, presence of a shared substitution in EF1α between PNG138 and EF1α can not be explained based on the available data. The generally high mitochondrial divergence indicates complex mechanisms are at work, and the mtDNA data are not necessarily the answer but rather a starting point for a population genetic study in its own right.

## Supplementary Material

XML Treatment for Carabdytes upin tindige
